# Practical Considerations in Gene Therapy for HIV Cure

**DOI:** 10.1007/s11904-013-0197-1

**Published:** 2014-01-22

**Authors:** Rodica Stan, John A. Zaia

**Affiliations:** Department of Virology, Beckman Research Institute of City of Hope, 1500 East Duarte Road, Duarte, CA 91010 USA

**Keywords:** HIV/AIDS, HIV, Gene therapy, CD4 T cell, Hematopoietic stem progenitor cell (HSPC), Lentivirus, Vector, siRNA, Expense, Risk benefit analysis, HIV infection, HIV-1 replication, Cell-based approaches, HIV-1 reservoir, Challenges, Strategies

## Abstract

Despite the success of antiretroviral therapy in suppressing HIV-1 replication and extending the life of HIV-1 infected individuals, this regimen is associated with risks for non-AIDS morbidity and mortality, requires life commitment, and has a high cost. In this context, gene therapy approaches that have the potential to cure HIV-1 infection present a clear option for eradication of the virus in the next decades. Gene therapy must overcome concerns related to its applicability to HIV-1 infection, the safety of cytotoxic conditioning required for cell-based approaches, clinical trial design, selection of gene-modified cells, and the restrictive cost of manufacturing and technology. These concerns are discussed herein in the context of the most relevant gene therapy studies conducted so far in HIV/AIDS.

## Introduction

The ultimate goal of a gene-therapy strategy for the cure of HIV/AIDS is to contribute a new set of immune cells that would be resistant to HIV-1 infection and would reconstitute the immune system. The recovered immune function would then simultaneously control infection and destroy the endogenous viral reservoir. The widespread use of antiretroviral therapy (ART) has achieved partial success in this respect by controlling the HIV-1 infection in most individuals. ART improves the health and extends the life of HIV-1 infected individuals and reduces the rate of viral transmission from individual to individual. However, this regimen is associated with several comorbidities, requires strict compliance with a lifelong drug regimen, and has little impact on the elimination of the HIV-1 reservoir [1•]. Latently infected, resting CD4+ T cells have been shown to persist in HIV-1 infected individuals treated with ART who otherwise have minimal levels of virus in the plasma [2, 3••].

For the purposes of this discussion, it is assumed that use of gene-modified, autologous cell-based strategies have the potential to achieve the goal outlined above. The recent success in apparently curing HIV-1 infection using allogeneic cell-based approaches [4•, 5••, 6•], although remarkable, will not be considered here because of the expense and toxicity of this approach. Strategies using autologous CD4 T cells or hematopoietic stem progenitor cells (HSPC), although also complex, have a more direct path to the clinic in the non-malignant HIV/AIDS patient. In addition to these cell-based approaches, recent investigations have focused on (a) drugs that reactivate latent HIV-1 from resting CD4+ T cells prior to the therapeutic intervention to eradicate the virus [7], and (b) immune reagents, such as neutralizing antibodies [8, 9, 10••, 11•], which enhance the anti-HIV-1 immune responses. These strategies are attractive and could well be used adjunctively with cellular therapy, but they will not be discussed here.

Autologous gene therapy-based strategies, while equally challenging in terms of practical application, have the potential to be relatively safe and would provide either a sterilizing cure, by completely eliminating the HV-1 reservoir, or a functional cure, by enabling long-term control over the virus in the absence of ART (ie, durable drug-free remission) [12].

## Gene Therapy Challenges

Gene-therapy based approaches most commonly aim to create a robust HIV-1 resistant immune system by targeting viral or cellular elements necessary to suppress viral infection after transplantation of resistant T cells or HSPC. Such strategies ultimately protect progeny CD4+ T cells and other HIV-1 susceptible cells from further viral infection and lead to the restoration of the immune function. To achieve this goal, the transgene or a combination of genes of interest is cloned into a delivery vehicle, a viral, or nonviral vector, which is then delivered efficiently into the target cells (T cells or HSPC) ex vivo [13•, 14]. These genetically modified cells are then release tested for safety and quality control and then re-infused into the patient. Except for strategies whereby genetic modification occurs by transient exposure to nucleases [15•, 16], most gene therapy approaches require long-term stable expression of the transgene in modified cells. Integrating and nonintegrating vectors are used and continue to be developed, each having their own unique advantages and disadvantages [17, 18]. For example, integrating vectors deliver the transgene into host DNA with the promise of long-lived expression, whereas nonintegrating vectors have a shorter period of expression and thus minimize the theoretical risk of insertional mutagenesis [16]. Finally, engraftment of the gene-modified cells is a pre-requisite for the success of this approach, which most likely requires conditioning with chemotherapy or non-chemotherapy agents, prior to the transplant.

The field of gene therapy is making sustained efforts to design better strategies for efficient, safe and cost-effective gene delivery, to identify new molecular targets for improved antiviral effect, and to move toward clinical trials that test the safety and feasibility of these approaches (discussed in [13•, 19]).

## HIV-1 Gene Therapy Strategies

A diverse array of transgenes to suppress HIV-1 functions or block the infectious cycle belong mostly to 2 groups of suppressors: nucleic acid-based and protein-based (reviewed in [20]). Current gene therapy strategies use combinations of different types of transgenes with viral and/or cellular targets that aim to completely neutralize HIV-1 functions at specific points in the infection cycle. In contrast to targeting a single step of HIV-1 infection, the advantage of inhibiting a number of critical steps in virus replication is that complete and effective suppression is more likely to achieve. In addition, use of combinations of different types of transgenes is also expected to prevent the emergence of HIV-1 variants that escape the gene-based strategy. A summary of published gene therapy clinical trials using these approaches is included with more details and references in Table 1.

**Table 1:** Summary of published clinical trials of anti-HIV gene therapy (modified from [20])

Mechanism	Target	Delivery of genes/cell transplantation	Phase	References
HSPC-based studies				
RNA decoy	Viral (rev protein)	Retroviral (MMLV) into autologous CD34+ HSPC	Pilot	[21]
Ribozyme	Viral (tat-vpr mRNA)	Retroviral (MMLV) into autologous CD34+ HSPC	I-II	[22••, 23, 24]
shRNA RNA decoy (TAR) ribozyme	Viral (tat-rev mRNA) Viral (tat protein) Host (CCR5 mRNA)	Lentiviral vector (SIN HIV) into autologous CD34+ HSPC	Pilot	[25••]
Transdominant negative Rev mutant	Viral (Rev protein)	Retroviral MoMLV-based vector into allogeneic CD34+ HSPC	Pilot	[26]
T-cell-based studies				
Inhibitory Rev10 protein	Viral (Rev protein)	Plasmid or retroviral –based vector into autologous T cells	Pilot	[27, 28]
HIV-specific T cells	CD4zeta chain	Retroviral (MMLV-based) vector into autologous CD4 and CD8 cells	II	[29]
Ribozyme	Viral (U5 and pol mRNA)	Retroviral (MMLV) into autologous CD4+ T cells	I	[30]
C46 peptide	Viral (env protein)	Retroviral (MMLV) into autologous CD4+ T cells	I	[31]
Ribozyme	Viral (tat-vpr mRNA)	Retroviral (MMLV) into syngeneic CD4+ T cells	Pilot	[32, 33]
Antisense	Viral (env mRNA)	Lentiviral vector (LTR HIV) into autologous CD4+ T cells	I-II	[34]
Antisense	Viral (env mRNA)	Lentiviral vector (VRX496-T; trade name, Lexgenleucel-T) into autologous CD4+ T cells	I-II	[35•]
Antisense	Viral (TAR and/or Rev)	Retroviral vector into uninfected lymphocytes from twin donor	I-II	[36]

### RNA-Based Suppressors, Including RNA-Antisense, RNA Interference (RNAi), Ribozymes, and RNA Aptamers (Reviewed in [37, 38]

Newly transcribed RNA-antisense molecules bind to mRNA sequences and prevent translation of the encoded protein, thereby resulting in loss or reduction of gene function. In one of the earliest lentiviral-based gene therapy studies against HIV-1 infection, an antisense mRNA to HIV-1 *env* expressed from a lentiviral vector was safely used in the setting of T-cell immunotherapy [34]. Further studies with the product (VRX496-T; trade name Lexgenleucel-T) given as multiple infusions were performed more recently in patients with well-controlled HIV-1 infection [35•]. The antiviral effect of the product was evaluated in a subset of treated patients who underwent an analytical treatment interruption (ATI) of their antiviral medication, the idea being that the ATI allows selection of the gene-modified cells once the virus is again present in the plasma. The study demonstrated a reduction in the viral load and found that VRX496-T put antisense-mediated genetic pressure on the virus during infection.

RNA decoy molecules attempt to compete with specific HIV-1 RNA elements that bind viral proteins as part of the replication cycle by overexpressing their RNA homologs. TAR (TAT response element) and RRE (Rev-response element) are 2 such cis-acting factors that are necessary for proper function of the key HIV-1 regulatory proteins, Tat and Rev. These elements have been evaluated in clinical trials, 1 in transduced and transplanted marrow-derived hematopoietic stem cells in HIV-1 infected children [21] and 1 in CD34+ HSPC transduced and transplanted in HIV-1 infected patients with Non-Hodgkin’s Lymphoma (NHL) who required autologous transplantation [25••].

Ribozymes are RNA molecules that can cleave RNA targets at specific sequences and thus can be used to inactivate HIV-1 RNAs [17]. A ribozyme has been used as a component of combinatorial gene therapy in HIV-1 infected patients with NHL mentioned above [25••].

RNAi, which naturally occurs in a variety of organisms, including fungi, plants, insects, protozoans and mammals [39], is based on a cellular process, in which double-stranded RNA (dsRNA) induces a post-transcriptional degradation of homologous transcripts. Several studies have demonstrated that the functional unit of RNAi, the siRNAs, can elicit sequence-specific target downregulation [40]. The successful expression of siRNAs in mammalian cells has allowed RNAi to be applied as a potent mechanism for inhibition of HIV-1 infection and anti-HIV therapy [41, 42]. The siRNA can be used as an antiviral agent either by transfection of the preformed siRNAs [43] or by intracellular expression of siRNAs. The latter approach has been utilized in a gene therapy setting, providing anti-HIV-1 RNAs to hematopoietic cells susceptible to HIV-1 infection [25••].

### Protein-Based Suppressors

Normal viral functions can be suppressed by expression of transdominant mutant proteins that act as competitors of cognate HIV-1 proteins. The most experimentally advanced transdominant protein to date is a mutant Rev protein (RevM10) [44], which retains 2 Rev functions: the ability to bind RRE on the viral genome and the ability to form Rev multimers. However, because RevM10 cannot exert its regulatory role in transporting unspliced or singly spliced RNAs from the nucleus to the cytoplasm, susceptible cell lines that express RevM10 exhibit long-term resistance to HIV-1 replication (>30 days). Human CD34+ blood progenitor cells transduced with RevM10 can give rise to T lymphocytes that exhibit significant resistance to challenge with HIV-1 [26].

## Clinical Trials of Gene Therapy Using Autologous T Cells

One cell-based strategy for gene therapy against HIV-1 infection targets autologous T lymphocytes, including CD4+ or CD8+ T cells [27, 34]. Mature T lymphocytes are easily harvested from the peripheral blood of donors and can be expanded to large numbers in vitro using cell surface stimulation with antibodies to the markers CD3 and CD28 [45]. Targeting mature T lymphocytes for genetic modification has other advantages, which make it the method of choice for initial evaluation of such gene therapy strategies. The effect of the therapeutic gene on cell survival, viral load, and other parameters can be rapidly evaluated. Autologous T cells can be transduced and immediately selected in vitro using a marker gene included in the vector, so that the reinfused population contains a high percentage of genetically modified cells.

Several clinical trials have evaluated gene therapy products against HIV-1 infection using autologous T lymphocytes (Table 1).

The first clinical study of a gene therapy approach in HIV-1 infected individuals evaluated the ability of RevM10 to extend the survival of transduced CD4+ T cells in vivo [27]. Despite showing limited and transient duration of engraftment, the study was followed by another one, which evaluated a retrovirus delivery vector for RevM10. Survival of T cells expressing RevM10 was improved relative to that of T cells transduced with a negative control vector [28] (Table 1). A recent study of conditionally-replicating lentiviral vector expressing a long antisense to HIV-1 targeting HIV-1 *env* showed that CD4 T cells (VRX496-T) have been safely infused into HIV-1-infected patients with well-controlled viremia (discussed above [35•]). Autologous transfer of these cells was safe in chronic viral infection and 6 out of 8 patients who underwent ATI experienced a significant decrease in viral load (*P* = 0.08).

Gene therapy strategies that aim to prevent or disrupt expression of chemokine receptors that facilitate HIV-1 entry into the target cell are particularly attractive. They obstruct the very first step in HIV-1 infection (ie, entry into the target cell), rather than blocking a viral event that occurs after the establishment of the proviral DNA into the cellular genome. The survival advantage of chemokine receptor-modified cells has been demonstrated in animal studies [15•], and has started to demonstrate survival advantages in vivo in HIV-1-infected individuals [46]. Clinical studies attempting to mimic the impact of the natural CCR5 deletion on HIV-1 infection are ongoing (NCT01252641 and NCT01044654). These studies evaluate CD4+ T cells modified using zinc finger nucleases to edit the CCR5 gene [16] and successful impact of this gene-therapy approach has been recently announced [46].

## Clinical Trials of Gene Therapy Using Autologous HSCT

Despite the demonstration of HIV-1 cure using allogeneic hematopoietic stem cell transplant (HSCT) [4•, 5••, 6•], the complexity of the method, including the requirement for tissue matching, the prolonged use of immunosuppressive medications to suppress graft vs host disease, and the cost, severely limits its current use. Autologous HSCT, if successful, would restore and maintain CD4 levels after a single treatment, and this has motivated the search for a method using genetically-modified HSPC (HSPC-GT). Two landmark studies of this type have been discussed, namely those of Mitsuyasu et al [22••] and DiGiusto et al [25••] (Table 1). These studies established feasibility, but showed that new methods were needed if levels of gene modification likely to have an anti-HIV-1 effect were to be attained.

The field is now at the point where the first use of busulfan-based conditioning therapy will be used in HIV/AIDS patients. A Calimmune-sponsored trial (NCT01734850) will use a lentivirus vector, encoding 2 HIV-1 entry inhibitors, to transduce both autologous CD4+ T cells and HSPCs in patients who are unable to continue ART due to intolerance or treatment fatigue. The study will test the safety and efficacy of no busulfan vs low dose (4 mg/kg) and high dose (8 mg/kg) busulfan used as conditioning therapy. A study from City of Hope (NCT01961063) will test the triple anti-HIV-1 lentivirus used by DiGiusto et al [25••] to determine if a busulfan regimen can be safely used in HIV/AIDS after successful treatment for non-Hodgkin lymphoma. Finally, a study from the Fred Hutchinson Cancer Research Center (NCT01769911) will test whether HSCT-GT, modified to contain an entry inhibitor, can be combined with a methylguanine methyltransferase [MGMT]-based selection method to expand gene-modified progeny cells.

## Obstacles Preventing the Application of Gene Therapy to Curing HIV-1 Infection

There are serious barriers that need to be overcome if gene-therapy is to succeed. The main obstacles that currently prevent the application of gene therapy to curing HIV-1 infection are (a) the difficulty of applying a rare disease therapy, such as gene transfer, to the large AIDS population; (b) resolving the safety of cytotoxic conditioning required for cell-based gene therapy; (c) clinical trial design issues, including: 1, correct identification of the target population based on thorough risk-benefit analysis, and 2, the lack of validated cell-based endpoints defining efficacy; (d) development of a safe method for selection of gene-modified cells; and (e) the restrictive cost of manufacturing and technology.

### Application of a Therapy Originally Developed to Treat Rare Diseases (ie, Gene Therapy, to a Large Volume Problem, which is the Widespread Infection with HIV-1)

Gene therapy was conceived to treat rare diseases having invariable poor outcome, such as severe combined immunodeficiency (SCID), chronic granulomatous disease (CGD), thalassemia, storage diseases, and other inborn errors of metabolism. It was not until its potential use in “intracellular immunization” was recognized [47] that gene therapy was applied to infectious diseases. Sophisticated processes required for gene therapy, such as efficient stem cell mobilization and collection by leukapheresis, vectored gene delivery, and cell transduction, are complex and expensive. Yet, these technical and financial barriers are accepted in treatment of rare diseases, where few other options are available, given the potential for cure. But the question remains as to whether similarly complex and costly therapy can be applied to the large population of persons with HIV-1, many of whom have restricted access to healthcare.

Lymphoma is probably the best example of a disease that faced similar hurdles. In this disease, autologous HSCT [aHSCT] is successfully used, but only when chemotherapy fails or risk factors suggest a need for HSCT, and optimally when debulking of disease is possible. When first proposed, aHSCT was considered too technically challenging and expensive for wide application, but today aHSCT can be an outpatient procedure, and there are an estimated ~30,000–35,000 procedures being performed worldwide every year [48]. More importantly, aHSCT will be curative in ~70 % of lymphoma patients. Admittedly, at present, aHSCT is not an economically viable solution to the general problem of HIV/AIDS. But it is foreseeable that with an eventual HIV-1 vaccine [10••], when the incidence of HIV-1 infection has declined perhaps to levels similar to lymphoma, aHSCT will be not only feasible but desirable as a curative procedure for this disease.

### Resolving the Requirement for Cytotoxic Conditioning for Cell-Based Gene Therapy

The first use of a conditioning regimen in patients receiving gene-modified aHSCT was performed in the setting of salvage therapy of AIDS-related lymphoma [25••]. In this pilot clinical study, patients undergoing high-dose chemotherapy and aHSCT were infused with both gene-modified (aHSCT-GT) and non-modified HSPC. The study demonstrated the safety and feasibility of the approach following myeloablative conditioning, but clearly such a potentially lethal approach could not be applied to HIV/AIDS patients without lymphoma/leukemia.

An optimized conditioning regimen needs to be determined and used for delivery of HSPC-GT for treatment of HIV/AIDS. This regimen must balance safety in a nonmalignant population with the requirement for efficient engraftment. Busulfan-based conditioning regimens have been used in clinical trials of gene therapy for the correction of human genetic diseases, including adenosine deaminase deficiency SCID [49, 50•] and X-linked CGD [51]. The safety of busulfan in HIV/AIDS patients remains to be determined in 2 currently active trials (NCT01734850 and NCT01961063). For CD4-based gene therapy, cytoreductive chemotherapy is also under study, using cytoxan as the pre-conditioning therapy (NCT01543152).

### Clinical Trial Design Issues for Gene Therapy Strategies Treating Chronic HIV-1 Infection

It is likely that the newly diagnosed HIV-1 infected population will dichotomize into a group of early treated patients who do very well and mimic elite nonprogressors and those with late AIDS diagnosis who do much less well. We know that currently a large proportion of individuals get tested for HIV-1 infection and start ART very late after initial exposure to the virus [52]. A subset of this latter group will do poorly because of reduced compliance [53]. It is this chronically infected patient population, having AIDS-related co-morbidities or ART-related toxicities, which will be initially targeted for gene therapy approaches. However, targeting the most relevant patient population, with the most balanced risk:benefit ratio in gene therapy clinical trials, is a serious challenge [54].

The population of chronically HIV-1 infected individuals ranges from poor responders, to ART-intolerant patients, to those with treatment fatigue, and those with AIDS-related malignancy. Each of these subpopulations has a risk:benefit balance and thus a varying justification for gene therapy approaches (Table 2). The principle of justice, as outlined in ethical discussions of clinical research, implies that the population at risk be the eventual population that would be treated should the therapy be successful. Thus, the HIV-1 lymphoma population, although used initially as an ideal design for testing novel gene therapy, is appropriate. But this group is less than optimal for HIV/AIDS gene therapy for several reasons. At a practical level, patients receiving salvage therapy are becoming less available due to improvements in front-line therapy [55]. However, once in remission, these patients are at some risk for myelodysplasia and secondary malignancy due to the completed anti-lymphoma therapy, and this could bias the observations needed to confirm research treatment safety. Also, although useful as a test-of-concept, the results from the HIV-related lymphoma population may not necessarily translate to the treatment of other HIV/AIDS patients targeted for studies testing gene therapy products and non-myeloablative conditioning regimens.

**Table 2:** Potential target populations for evaluation of gene therapy-based approaches (modified from [54])

Target population	HIV-1 subgroup	Risk vs benefit: appropriate?	Ready availability	Relevant to product development
Optimal ART therapy	HIV-1+ on cART HIV load: low CD4 count: high	No	No	Possibly
Off ART therapy	HIV-1+ off cART HIV load: high CD4 count: high	Yes	Yes	Ideal population to observe efficacy
HIV infected, immune nonresponder (INR) on ART	HIV-1+ on cART HIV load: low CD4 count: low	Yes	Yes	INR Population suited for needed therapy
AIDS malignancy	HIV-1+ on cART With NHL HIV count: low CD4 count: low or high	Yes	No	Can show test-of-concept, but less applicable for translation to general AIDS population

The endpoint of anti-HIV-1 trials require an antiviral effect measured by HIV-1 RNA plasma levels for demonstration of efficacy. This can usually be done with relatively short duration studies. But what if the therapy provides reconstitution of CD4 counts or has an effect on the HIV-1 reservoir that takes months to years? For example, the recent studies with gene modified autologous CD4+ T cells using a zinc finger nuclease to disrupt the CCR5 gene have reported a decrease in HIV-1 proviral DNA at 12 months [46]. Admittedly, a profound effect on the reservoir should affect the plasma HIV-1 RNA level, but these studies are usually performed while the research participant remains on ART. Thus, the cell-based effects could occur without an observable effect on HIV-1 RNA plasma levels.

Demonstrating an effect on surrogate markers of outcome, eg, restoration of CD4 count and reversal of inflammation markers, will therefore be the challenge [18, 56]. This raises the question of whether definition of efficacy in the context of gene therapy for HIV-1 cure will need to be redefined as stabilization of CD4+ T-cell count with or without eradication of HIV-1 reservoir.

### Development of a Safe Method for Selection of Gene Modified Cells

The HSPC-GT trial of DiGiusto et al [25••], although safe using lentivirus transduced HSCT, was used with nontransduced HSCT for ethical reasons related to lymphoma salvage therapy, and resulted in a level of marking that was less than 0.35 % of the progeny PBMC. This low level of marking points out the major limitation with the use of aHSCT-GT, which is the requirement for engraftment of adequate numbers of modified stem cells to overcome the endogenous pool of stem cells. In addition, these types of first-in-human studies in HIV/AIDS require a back-up unit of HSPC to manage potential hematopoietic complications, which places a further practical challenge on the study to collect sufficient stem cell numbers.

Thus, since non-myeloablative aHSCT-GT will always face the problem of competing endogenous stem cells, a method for selection of protected cells is necessary. Several studies have suggested that HIV-1 itself could be used for selection [15•, 57••, 58], and patients have undergone ATI [22••, 35•, 46] for the purpose of selecting gene-modified cells using the selective pressure put by the actual virus. An alternative is the use of chemical selection, for example with MGMT, which has been used in humans with glioblastoma [59] and has recently been shown to protect macaques from SIV infection [57••]. This approach is moving rapidly to clinical testing in HIV/AIDS [60–62], as there is also evidence from animal studies that HIV-1 can induce an expansion of gene-modified cells.

**Fig. 1:**
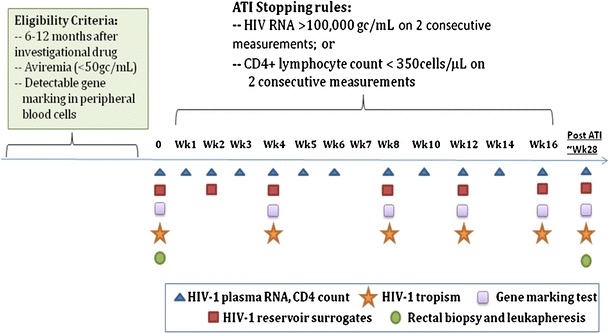
Sample of schema for ATI design in anti-HIV gene therapy trials

Of course, in addition to providing the means for virus-based selective pressure, ATI is used to determine if a new treatment actually has antiviral activity [22••]. The aHSCT-GT trial of Mitsuyasu et al was the first Phase 2 trial of an aHSPC-GT-based therapy in HIV/AIDS and provided a novel design for testing efficacy using ATI. The safety of ATI is critical, and an example of appropriate design for such treatment is shown in Figure 1. The ATI should not begin prior to demonstrating evidence that a minimal, pre-determined level of CD4 gene modification has been achieved. Then, during the ATI, participants should be monitored closely and with stopping rules based on changes in HIV-1 RNA level in plasma, significant reduction in CD4 counts, and clinical signs and symptoms of viremia.

### The Restrictive Cost of Manufacturing and Availability of Technology

The high cost associated with gene therapy, whether T-cell- or HSPC-based, challenges the economic viability of this approach. As with aHSCT for lymphoma, in which high initial costs were lowered with experience in the method, it is likely that aHSCT-GT will be inordinately expensive at first and then, as the cost of goods decreases, it will become affordable. At present, it is the cost of goods and services that contributes the major portion of cost to aHSCT-GT, and when there are more providers of these goods, they should become less expensive. In the current context of HIV-1 treatment, where the cost of lifelong ART therapy is estimated to be $420,000–$755,000 USD per individual, with 73 % of the cost going toward ART [63, 64], and even the conversion to generic once daily ART, estimated to only reduce lifetime costs by ~ $42,000/patient [65], successful gene therapy for HIV-1 could be cost effective [18, 54].

This obviously would be driven by the efficacy of the therapy, but if aHSCT-GT could favorably impact outcome in those HIV/AIDS patients with increased health risks, then aHSCT-GT could result in a significant reduction of the AIDS-related healthcare spending. Admittedly, this is a significant hurdle, but a one-time intervention with a gene therapy approach, which is practical in implementation and could cure HIV-1 infection, would be a viable alternative, both economically and medically.

## Conclusions

Despite the sustained success of ART against HIV-1 infection and the significant improvements made to this regimen, as long as it must be taken daily for a lifetime, there will be a desire to cure HIV-1 infection using a single treatment. It is precisely in this aspect—of showing an impact on the viral reservoir and, therefore, potential for a cure—that the dream of gene therapy continues. Better anti-HIV targets and improved vectors, delivery systems, and cellular processing will contribute to the continued progress of gene therapy approaches against HIV-1 infection. Ideally, gene therapy will eventually rely on a single injection of genetic material that will prevent or control HIV-1 infection. A delivery system, which would result in production of protective humoral immunity, might ultimately fulfill the promise of intracellular immunization [47]. With current breakthroughs [66••, 67], antibodies that recognize and block evolutionarily conserved, but essential structures of the HIV viral envelope, could reinvigorate the search for antibody based HIV vaccines [10••]. Current progress with neutralizing antibodies administered with novel gene delivery highlight the applicability of gene therapy strategies in curing HIV-1 infection [9, 68–70]. One can also anticipate new multiplexed anti-HIV-1 vector combinations that include host restriction factors [71], directed expression of HIV-1 neutralizing antibodies [11•], as well as improved conditioning regimens for T cell and HSPC gene transfer.
